# Association of obesity and anovulatory infertility

**DOI:** 10.31744/einstein_journal/2020AO5150

**Published:** 2020-02-27

**Authors:** Valéria Fichman, Roseli de Souza Santos da Costa, Teresa Cristina Miglioli, Lizanka Paola Figueiredo Marinheiro

**Affiliations:** 1 Fundação Oswaldo Cruz Rio de JaneiroRJ Brazil Fundação Oswaldo Cruz , Rio de Janeiro , RJ , Brazil .; 2 Universidade Iguaçu IguaçuRJ Brazil Universidade Iguaçu , Iguaçu , RJ , Brazil .

**Keywords:** Infertility, female, Anovulatory, Obesity, Reproduction, Feeding behavior, Motor activity

## Abstract

**Objective:**

To verify the association of obesity and infertility related to anovulatory issues.

**Methods:**

This case-control study was carried out with 52 women, aged 20 to 38 years, divided into two groups (infertile − cases − and fertile − control), seen at outpatient clinics, in the period from April to December, 2017.

**Results:**

We found significant evidence that obesity negatively affects women’s fertility (p=0.017). The group of infertile women was 7.5-fold more likely to be obese than fertile women.

**Conclusion:**

Strategies that encourage weight control are indicated for women with chronic anovulation, due to hight metabolic activity of adipose tissue.

## INTRODUCTION

Infertility is defined as the absence of pregnancy after one year of regular sexual intercourse without the use of contraceptives, for women under 35 years, and as of the sixth month of attempting to conceive for women 35 or more years of age. ^( 1 )^ It is an ever more frequent phenomenon in developed societies and affects about 48.5 million couples around the world. ^( 2 )^

Fertility can be negatively affected by various hypothalamic, pituitary, thyroid, adrenal, and ovarian disorders, as well as by the usage of drugs, advanced age, and obesity. ^( 3 )^ Among the primary factors involved in infertility of couples, those related to female issues are classified as being tuboperitoneal and ovulatory. ^( 4 )^ The latter are influenced by extremes of body weight, which contribute towards insulin resistance, ^( 5 )^ reflected in anovulation. ^( 6 )^

It is increasingly acknowledged that the obesity epidemic contributes towards fertility problems. ^( 7 )^ According to information from the *Pesquisa de Orçamentos Familiares* [Family Budget Research], performed in Brazil between 2008 and 2009, among women aged 20 or more years, 48% were overweight (body mass index/BMI ≥25kg/m ^2^ ) and 17% were obese (BMI ≥30kg/m ^2^ ). This is a worldwide issue. ^( 8 )^ Until now, there have been few observational studies about the association between obesity and infertility in the Brazilian population.

The lack of knowledge about infertility hinders the adoption of preventive measures in its treatment. ^( 9 )^ Obesity, and primarily abdominal obesity, participates in this etiology, and the awareness of its relation with infertility is extremely important, so that the nutritional status of subfertile women, that is, women with absence of conception who actively want to conceive, can be corrected. ^( 10 )^

Ovulatory defects and inexplicable causes represent more than 50% of etiology of infertility ^( 11 )^ and, although not the only factors involved, one should carefully analyze the ovulatory issues with the intention of positive reversal for a fertility process.

Abdominal obesity, common in patients with polycystic ovary syndrome (PCOS), is involved in the secretion of various hormones and cytokines that contribute towards the start of a proinflammatory status and oxidative damage, ^( 12 )^ negatively affecting the complex hormone environment, generating disorders in the hypothalamus-pituitary-ovarian axis ^( 13 )^ and, consequently, acting in the deregulation of the menstrual cycle and in the reproductive capacity of the woman.

Polycystic ovary syndrome is a heterogeneous condition characterized by irregular ovulations or anovulation, hyperandrogenism, oligomenorrhea, and subfertility. ^( 14 )^ Obesity occurs in 30% to 75% of women with PCOS, ^( 15 )^ and increases the magnitude of hormone and metabolic dysfunction of these women. ^( 14 )^

## OBJECTIVE

To investigate the association of obesity and infertility related to anovulatory issues in women, and to identify associated factors.

## METHODS

### Study population

This is a case-control study conducted with women seen at outpatient clinics of the *Instituto Nacional da Saúde da Mulher, Criança e do Adolescente Fernandes Figueira/Fundação Oswaldo Cruz* (IFF/Fiocruz), in the city of Rio de Janeiro (State of Rio de Janeiro/RJ), between April and December 2017. Pairing of the sample was done by age.

As cases, women treated at the infertility outpatient clinic, with anovulatory problems defined by the physician in charge, were enrolled, and as controls, pregnant women seen at the prenatal outpatient clinic, who desired to participate in the study. In both groups, women under 20 years of age or over 38 years of age were excluded, in addition to those seen at the infertility outpatient clinic with tuboperitoneal problems or their partners’ sperm issues.

The parameters used for the diagnosis of anovulatory issues included clinical history and determination of serum levels of follicle-stimulating hormone (FSH), luteinizing hormone (LH), estradiol and progesterone.

The sample was calculated considering the results of the infertility study, ^( 16 )^ which observed the marker of oxidative stress in follicular fluid in infertile women (lipid peroxidase/LPO). In Group 1, women without PCOS, with abdominal obesity (a value of 1.0±0.3) were enrolled, and for Group 2, women without PCOS and without abdominal obesity (a value of 0.79±0.2).

The confidence interval adopted was 95% with a power of 80%, generating a sample size of 24 observations in each group, totaling up 48. The sample was incremented only in the controls by 16% (4 cases), due to the fact of more patients not having been seen with anovulation at the infertility outpatient clinic during the chosen period for collection, totaling 52 participants in the study, classified as Case (24) and Control (28) Groups.

### Measurements and data analysis

Data collection was performed using the International Physical Activity Questionnaire (IPAQ) and a previously tested questionnaire, prepared for the investigation, divided into three parts: the first contained questions related to sociodemographic information, such as age, occupation, district, and city of residence. The second part contained information on the clinical history, such as *diabetes mellitus* , hypertension, PCOS, other diseases, as well as the habit of smoking and using alcoholic beverages. The use of alcoholic beverages was classified as 1 - does not use / 2 - uses once in a while / 3 - uses daily / 4 - uses on weekends.

In the third part, the anthropometric values of weight, height, abdominal circumference (AC), and hip circumference (HC) were described. The BMI was categorized as 1 - low weight: <18.5kg/m ^2^ ; 2 - adequate: 18.5 to 24.9kg/m ^2^ ; 3 - overweight: 25.0 to 29.9kg/m ^2^ ; 4 - obesity: ≥30.0kg/m ^2^ .

The predictive variables analyzed in this study were BMI, physical activity, and alcohol and smoking habits. The remaining variables cited above, such as AC, HC, *diabetes mellitus* , hypertension, and PCOS were included in the study but were not analyzed between the two groups.

The BMI was calculated for each woman, by means of the weight/height squared ratio, and was classified as per the criteria of the World Health Organization (WHO), 2000 (https://www.who.int/nutrition/publications/obesity/WHO_TRS_894/en/).

The anthropometric measurements of weight, height, AC, and HC of the cases were checked on the days patients were seen at the infertility outpatient clinic, and the anthropometric measurements of weight and height were obtained from pregestational reports of weight and height from the controls at the time of the interviews. For pregnant women who did not know their height, it was measured at the time of the interview.

The AC of the cases was measured around the region of the greatest diameter, normally coinciding with the umbilical scar. ^( 17 )^

The AC and HC variables were not evaluated for the Control Group, due to the anatomical modification related to pregnancy.

The variables of use of alcohol, smoking, and physical activity were collected by self-reports and checked relative to the pregestational period in the Control Group, and relative to the current period for the Case Group. The condition of having *diabetes mellitus* , hypertension, and PCOS was only checked in the Case Group.

The IPAQ was proposed by WHO in 1998 and was validated in Brazil by means of a pilot study in Brazilian young adults. ^( 18 )^ We used the short version, which covers the types of physical activity that people do in their daily living, as part of a large study divided among different countries.

The data was analyzed based on frequency (categorical variables), mean, and standard deviation (numerical variables).

For comparison between the groups, the χ ^2^ and Fisher’s exact tests were used, carried out with (SPSS) software, version 22.

### Ethical considerations

All the participants signed the Informed Consent Form (ICF). The research project was approved by the Committee of Ethics and Research in Human Beings (CEPIFF), under opinion number 2.374.634, CAAE: 63617616.6.0000.5269, submitted on February 15, 2017.

## RESULTS

The mean age of the infertile women observed was 31 years, and of fertile women, 27 years.

The hypothesis test of the women seen at the infertility and prenatal outpatient clinics of IFF/Fiocruz by means of the variable BMI, presented with statistical evidence (p<0.05) that obesity interfered in fertility of women. Table 1 shows the distribution of cases and controls, according to the variables studied.

**Table 1:** Comparison of the characteristics of women of two groups studied

Patients	Case Group (n=24) %	Control Group (n=28) %	p value
BMI			0.017
Low weight and eutrophy	17	54	
Overweight	33	25	
Obesity	50	21	
Uses alcoholic beverages			0.615
No/once in a while	96	89	
Daily/Weekends	4	11	
Smoking			0.115
No	100	86	
Yes	0	14	
Physical activity			1.000
Very active/active	58	61	
Irregularly active/sedentary	42	39	

Results expressed as %. BMI: body mass index.

Of all the variables with p<0.05 that were entered into the multivariate model, only BMI demonstrated significance; the Case Group was 7.5-fold more likely to be obese when compared to the fertile women ( Table 2 ).

**Table 2:** Odds ratio for cases and controls, as per body mass index

BMI	Odds ratio
Low weight and eutrophy	-
Overweight	1.75 (0.43-7.2)
Obesity	7.5 (1.72-32.8)

BMI: body mass index.

As to being a smoker, the comparison between the groups was close to statistical significance (p=0.115), although no infertile patient smoked, and only four women from the prenatal outpatient clinic smoked (14%). As to alcoholic beverages, in both groups, the majority of women did not consume them; no woman in the Case Group used alcohol daily, and only two fertile patients (7%) consumed alcoholic beverages every day.

Active and irregularly active women were the majority in both groups studied.

Abdominal obesity was noted in 21 infertile patients (87.5%), indicating an increased risk of metabolic complications in these patients ( Table 3 ).

**Table 3:** Risk of metabolic complications in the cases

Cut-off point/classification	
AC <80cm/no risk	12.5
AC ≥80cm/with increased risk	87.5

Results expressed as %. AC: abdominal circumference.

Of the infertile patients, only one reported being diabetic (4.2%), and two (8.3%) were hypertensive.

## DISCUSSION

This study investigated the association between obesity and infertility, two prevalent issues in modern female life, according to other findings already described in literature. ^( 5 , 7 , 8 , 14 , 16 )^ Knowledge about the association between obesity and infertility is important to deepen understanding of the factors that involve the worldwide obesity pandemic ^( 19 )^ and comprehend it in addition to chronic noncommunicable diseases.

Most of the women with excessive weight, that is, overweight or obesity, were in the Case Group. Observing 1,880 women seen at infertility clinics in the United States and Canada, a study concluded that the relative risk of anovulatory infertility is 3.1 times greater in obese women. ^( 20 )^ The relation between weight loss and improvement of the reproductive function is well established, but the mechanisms that act in optimizing this function need to be better clarified. ^( 21 )^

It is important to point out there are several factors that influence the anovulation process, and these often occur concomitantly: oxidative stress, which can affect the follicular fluid, ^( 16 )^ and modifications in the metabolism of sex hormones ^( 22 )^ and insulin resistance. ^( 23 )^

Obese women may present with damaged or decreased tolerance to glucose without being carriers of *diabetes mellitus* , since there is an intermediate stage between the homeostasis of glucose and diabetes. ^( 24 )^ Half of the cases studied presented with obesity, and 95.8% of these patients did not suffer from *diabetes mellitus* , suggesting that they could be in this deficient glucose tolerance stage.

As to the use of alcoholic beverages and smoking, these findings were different relative to those of other studies, ^( 3 , 9 , 25 )^ that observed an interference of these factors in the female reproductive capacity, since they cause modification in the hormone levels and decrease libido.

As to hypertension, 91.7% of cases studied reported not having this disease. Despite the fact that obesity is related to several comorbidities, such as hypertension, *diabetes mellitus* type 2, and some kinds of cancer and dyslipidemia, ^( 26 )^ there are obese patients that present with only one or none of these comorbidities. This does not mean that there are cases of healthy obesity, since the inflammatory process is inherent to this disease, causing various damages to the health of patients.

The result of physical activity, with few sedentary women (none in the Case Group and four – 14% − in Control Group), could be related to the manner in which the IPAQ classified the moderate activities, including domestic activities that moderately increase breathing rate or heart beats and were very often included by the participants of this study as the only physical activity reported. The potential of the present study was to demonstrate that population studies follow the same pattern found in studies carried out in other countries, ^( 7 , 11 )^ reinforcing the hypothesis that obesity interferes in infertility.

## CONCLUSION

Obesity influences infertility, that is, there is a positive correlation between obesity and infertility. Knowledge of such a relation is important in order to increase the chances of subfertile women of reversing the situation of infertility, and having a healthy pregnancy. Improving eating habits and engagement in physical exercises are important to promote changes in body composition and nutritional status of these women.

The best management of women with chronic anovulation should be the development of strategies that stimulate weight control, before beginning a treatment cycle for assisted reproduction.

We suggest hospitals providing treatment for infertility offer nutritional care and encourage the practice of physical exercise to the population seen.
