# Supplementary material for: Association of obesity and anovulatory infertility
Source: Einstein (Sao Paulo). 2020 Feb 27;18:eAO5150. doi: 10.31744/einstein_journal/2020AO5150 (PMC7053827; doi:10.31744/einstein_journal/2020AO5150)
Supplement: Translation

# Translation: Associação entre obesidade e infertilidade anovulatória

**Authors:** Valéria Fichman, Roseli de Souza Santos da Costa, Teresa Cristina Miglioli, Lizanka Paola Figueiredo Marinheiro

**Affiliations:** 1 Brasil Fundação Oswaldo Cruz, Rio de Janeiro, RJ, Brasil .; 2 Brasil Universidade Iguaçu, Iguaçu, RJ, Brasil .

**Keywords:** Infertilidade feminina, Anovulatória, Obesidade, Reprodução, Comportamento alimentar, Atividade motora

## Abstract

**Objetivo:**

Verificar em mulheres a associação entre obesidade e infertilidade relacionada a questões anovulatórias.

**Métodos:**

Estudo de caso-controle com 52 mulheres, de 20 a 38 anos, divididas em dois grupos (mulheres inférteis − casos − e férteis − controles), atendidas em ambulatórios, no período de abril a dezembro de 2017.

**Resultados:**

Verificou-se evidência significativa de que a obesidade afeta negativamente na fertilidade das mulheres (p=0,017). O grupo de mulheres inférteis teve 7,5 vezes mais chances de serem obesas quando comparadas às mulheres férteis.

**Conclusão:**

Estratégias que estimulem o controle do peso são indicadas para mulheres com anovulação crônica devido à elevada atividade metabólica do tecido adiposo.

## INTRODUÇÃO

A infertilidade é definida como a ausência de gravidez após 1 ano de relações sexuais regulares sem uso de contraceptivos, para mulheres com menos de 35 anos, e a partir do sexto mês de tentativa de concepção, para as com 35 ou mais anos de idade.^( 1 )^É um fenômeno cada vez mais comum nas sociedades desenvolvidas e afetando cerca de 48,5 milhões de casais em todo mundo.^( 2 )^

A fertilidade pode ser afetada negativamente por diferentes transtornos hipotalâmicos, pituitários, tireoideanos, adrenais e ovarianos, bem como pelo consumo de drogas, idade avançada e pela obesidade.^( 3 )^Dentre os principais fatores envolvidos na infertilidade conjugal, aqueles relacionados às questões femininas são classificados como os de tuboperitoneal e ovulatórios.^( 4 )^Estes últimos são influenciados por extremos de peso corporal, que contribuem para a resistência insulínica,^( 5 )^refletindo em anovulação.^( 6 )^

É cada vez mais reconhecido que a epidemia de obesidade contribui para problemas de fertilidade.^( 7 )^Segundo informações da Pesquisa de Orçamentos Familiares realizada no Brasil entre 2008 e 2009, dentre as mulheres com 20 ou mais anos de idade, 48% estavam com peso excessivo (≥25kg/m^2^de índice de massa corporal − IMC) e 17% estavam obesas (IMC ≥30kg/m^2^). Esta é uma questão de escala mundial.^( 8 )^Até o momento, há poucos estudos observacionais sobre a associação da obesidade e a infertilidade na população brasileira.

A falta de conhecimentos sobre a infertilidade dificulta a adoção de práticas preventivas em seu tratamento.^( 9 )^A obesidade, principalmente a abdominal, participa desta etiologia, e o conhecimento de sua relação com a infertilidade é de extrema importância, para que se possa atuar no estado nutricional das mulheres subférteis, ou seja, mulheres com falta de concepção e que ativamente desejam conceber.^( 10 )^

Os defeitos ovulatórios e as causas inexplicáveis representam mais de 50% da etiologia da infertilidade^( 11 )^e, embora não sejam os únicos fatores envolvidos, devem-se analisar as questões ovulatórias cuidadosamente, no intuito de reversão positiva para um processo de fertilidade.

A obesidade abdominal, comum nas pacientes com síndrome do ovário policístico (SOP), está envolvida na secreção de vários hormônios e citocinas, que contribuem para o início de um estado pró-inflamatório e dano oxidativo,^( 12 )^refletindo negativamente no complexo ambiente hormonal, gerando distúrbios no eixo hipotálamo-hipofisário-ovariano^( 13 )^e, consequentemente, atuando na desregularização do ciclo menstrual e na capacidade reprodutiva da mulher.

A SOP constitui condição heterogênea caracterizada por ovulações irregulares ou anovulação, hiperandrogenismo, oligomenorreia e subfertilidade.^( 14 )^A obesidade ocorre em 30% a 75% das mulheres com SOP,^( 15 )^e aumenta a magnitude da disfunção hormonal e metabólica dessas mulheres.^( 14 )^

## OBJETIVO

Investigar em mulheres a associação entre obesidade e infertilidade, relacionada às questões anovulatórias, e identificar os fatores associados.

## MÉTODOS

### População de estudo

Trata-se de estudo caso-controle desenvolvido com mulheres atendidas nos ambulatórios do Instituto Nacional da Saúde da Mulher, da Criança e do Adolescente Fernandes Figueira/Fundação Oswaldo Cruz (IFF/ Fiocruz), no Rio de Janeiro (RJ), de abril a dezembro de 2017. O pareamento da amostra deu-se por idade.

Como casos, foram incluídas mulheres atendidas no ambulatório de infertilidade com questões anovulatórias definidas pelo médico responsável e, como controles, gestantes atendidas no ambulatório do pré-natal, que desejaram participar do estudo. Em ambos os grupos, foram excluídas as mulheres até 20 anos ou com mais de 38 anos de idade, além daquelas atendidas no ambulatório de infertilidade com problemas tuboperitoneal ou questões de esperma do parceiro.

Os parâmetros usados para diagnóstico das questões anovulatórias incluíram história clínica e avaliação das dosagens séricas dos hormônios folículo-estimulante (FSH), luteinizante (LH), estradiol e progesterona.

A amostra foi calculada considerando os resultados de estudo sobre infertilidade,^( 16 )^que observou o marcador de estresse oxidativo no fluido folicular de mulheres inférteis (peroxidase lipídica/LPO). No grupo 1, foram incluídas mulheres sem SOP, com obesidade abdominal (um valor de 1,0±0,3) e para o grupo 2, mulheres sem SOP e sem obesidade abdominal (um valor de 0,79±0,2).

Consideraram-se nível de confiança de 95% e poder de 80%, gerando tamanho amostral de 24 observações em cada grupo, com total de 48. A amostra foi incrementada apenas nos controles em 16% (4 casos), pelo fato de não terem sido atendidas mais pacientes com anovulação no ambulatório de infertilidade no período estipulado para a coleta, totalizando 52 mulheres participantes do estudo, classificadas nos Grupo Caso (24) e Controle (28).

### Medidas e análise dos dados

A coleta dos dados foi realizada utilizando-se o Questionário Internacional de Atividade Física (IPAQ) e questionário previamente testado, construído para a pesquisa, dividido em três partes: a primeira continha perguntas relacionadas a informações sociodemográficas, como idade, profissão, bairro e cidade onde reside. A segunda parte continha informações sobre a história clínica, como possuir *diabetes mellitus* , hipertensão arterial, SOP, outras doenças, hábito de fumar e de usar bebida alcóolica. O uso de bebida alcóolica foi classificado em: 1 - não usa / 2 - usa eventualmente / 3 - usa diariamente / 4 - usa final de semana.

Na terceira parte, foram descritos os valores antropométricos de peso, estatura, circunferência abdominal (CA) e circunferência do quadril (CQ). O IMC foi categorizado em: 1 - baixo peso: <18,5kg/m^2^; 2 - adequado: de 18,5 a 24,9kg/m^2^; 3 - sobrepeso: de 25,0 a 29,9kg/m^2^; 4 - obesidade: ≥30,0kg/m^2^.

As variáveis preditoras analisadas neste estudo foram IMC, atividade física, hábito de usar bebida alcóolica e de fumar. As demais variáveis citadas anteriormente: CA, CQ, *diabetes mellitus* , hipertensão arterial e SOP foram incluídas no estudo, mas não foram analisadas entre os dois grupos.

O IMC foi calculado para cada mulher, por meio da relação do peso/estatura ao quadrado, sendo classificado a partir dos critérios de 2000 da Organização Mundial da Saúde (OMS) (https://www.who.int/nutrition/publications/obesity/WHO_TRS_894/en/).

As medidas antropométricas de peso, estatura, CA e CQ dos casos foram aferidas nos dias de atendimento no ambulatório de infertilidade, e as medidas antropométricas de peso e estatura dos controles foram obtidas de relato do peso e da estatura pré-gestacional no momento das entrevistas. Para as gestantes que desconheciam sua estatura, esta foi aferida no momento da entrevista.

A CA dos casos foi mensurada na região com o maior diâmetro, coincidindo normalmente com a cicatriz umbilical.^( 17 )^

As variáveis CA e CQ não foram avaliadas para o Grupo Controle, devido à alteração anatômica provocada pela gravidez.

As variáveis uso de bebida alcóolica, fumo e atividade física foram coletadas por autorrelato e averiguadas em relação ao período pré-gestacional no Grupo Controle, e em relação ao período atual para o Grupo Caso. A condição de possuir *diabetes mellitus* , hipertensão arterial e SOP foi verificada apenas no Grupo Caso.

O IPAQ foi proposto pela OMS em 1998 e validado no Brasil por meio de estudo piloto em adultos jovens brasileiros.^( 18 )^Utilizou-se a versão curta, que aborda os tipos de atividades físicas que as pessoas realizam em seu dia a dia, sendo parte de um grande estudo desdobrado em diferentes países.

Os dados foram analisados a partir de sua frequência (variáveis categóricas), da média e do desvio padrão (variáveis numéricas).

Para comparação entre os grupos, foram utilizados os testes do χ^2^e exato de Fisher, efetuados no software (SPSS), versão 22.

### Considerações éticas

Todas as participantes assinaram o Termo de Consentimento Livre e Esclarecido (TCLE). O projeto de pesquisa foi aprovado pelo Comitê de Ética e Pesquisa em Seres Humanos (CEPIFF), sob parecer 2.374.634, CAAE: 63617616.6.0000.5269, submetido em 15 de fevereiro de 2017.

## RESULTADOS

A média de idade das mulheres inférteis observada foi de 31 anos e das mulheres férteis de 27 anos.

O teste de hipótese entre as amostras das mulheres atendidas no ambulatório de infertilidade e de pré-natal do IFF/Fiocruz, por meio da variável IMC, apresentou evidência estatística (p<0,05) de que a obesidade interferiu na fertilidade das mulheres. A tabela 1 apresenta a distribuição dos casos e controles, segundo as variáveis estudadas.

**Tabela 1:** Comparação de características das mulheres entre os dois grupos estudados

Pacientes	Grupo Caso (n=24) %	Grupo Controle (n=28) %	Valor de p
IMC			0,017
Baixo peso e eutrofia	17	54	
Sobrepeso	33	25	
Obesidade	50	21	
Usa bebida alcóolica			0,615
Não/eventualmente	96	89	
Diariamente/final de semana	4	11	
Fuma			0,115
Não	100	86	
Sim	0	14	
Atividade física			1,000
Muito ativo/ativo	58	61	
Irregularmente ativo/sedentário	42	39	

Resultados expressos por %. IMC: índice de massa corporal.

De todas as variáveis com p<0,05 que entraram no modelo multivariado, apenas o IMC demonstrou significância, sendo que o Grupo Caso teve 7,5 mais chances de serem obesas quando comparadas as mulheres férteis ( Tabela 2 ).

**Tabela 2:** *Odds ratio* para casos e controles, segundo o índice de massa corporal

IMC	*Odds ratio*
Baixo peso e eutrofia	-
Sobrepeso	1,75 (0,43-7,2)
Obesidade	7,5 (1,72-32,8)

IMC: índice de massa corporal.

Quanto a ser fumante, a comparação entre os grupos ficou próxima da significância estatística (p=0,115), embora nenhuma paciente infértil fumasse, e apenas quatro do ambulatório do pré-natal fumavam (14%). Em relação à bebida alcóolica, nos dois grupos, a maioria das mulheres não a consumia; nenhuma mulher do Grupo Caso fazia uso diariamente e apenas duas pacientes férteis (7%) consumia bebida alcóolica todos os dias.

As mulheres ativas e irregularmente ativas foram a maioria nos dois grupos estudados.

Observou-se obesidade abdominal em 21 pacientes inférteis (87,5%), pelo resultado da aferição da CA >80cm, indicando risco aumentado de complicações metabólicas nestas pacientes ( Tabela 3 ).

**Tabela 3:** Risco de complicações metabólicas nos casos

Ponto de corte/classificação	
CA <80cm/sem risco	12,5
CA ≥80cm/com risco aumentado	87,5

Resultados expressos por %. CA: circunferência abdominal.

Das pacientes inférteis, apenas uma referiu ser diabética (4,2%) e duas (8,3%) hipertensas.

## DISCUSSÃO

Este estudo investigou a associação entre obesidade e infertilidade, duas questões prevalentes na vida feminina moderna, à luz de outros achados já descritos na literatura.^( 5 , 7 , 8 , 14 , 16 )^Conhecer a associação entre obesidade e infertilidade é importante para se aprofundar em questões que envolvem a pandemia mundial da obesidade^( 19 )^e entendê-la para além das doenças crônicas não transmissíveis.

As mulheres com excesso de peso, ou seja, sobrepeso ou obesidade estavam, em sua maioria, no Grupo Caso. Ao se observar 1.880 mulheres atendidas em clínicas de infertilidade nos Estados Unidos e Canadá, estudo concluiu que o risco relativo de infertilidade anovulatória é 3,1 vezes maior nas obesas.^( 20 )^A relação entre perda de peso e melhora da função reprodutiva é bem estabelecida na literatura, mas os mecanismos que atuam na otimização desta função precisam ser melhor elucidados.^( 21 )^

É importante destacar que há vários fatores que influenciam no processo de anovulação, e estes ocorrem, muitas vezes, concomitantemente: o estresse oxidativo, que pode afetar o fluido folicular,^( 16 )^e as alterações no metabolismo dos hormônios sexuais^( 22 )^e na resistência insulínica.^( 23 )^

Mulheres obesas podem apresentar tolerância à glicose prejudicada ou diminuída sem serem portadoras de *diabetes mellitus* , pois há um estágio intermediário entre a homeostase de glicose e o diabetes.^( 24 )^Metade dos casos estudados apresentou obesidade e 95,8% do total destas pacientes não eram portadoras de *diabetes mellitus* , sugerindo que poderiam estar nesse estágio de tolerância à glicose prejudicada.

Quanto ao uso de bebida alcóolica e fumo, estes achados foram diferentes em relação aos de outros estudos,^( 3 , 9 , 25 )^que observaram interferência destes fatores na capacidade reprodutiva feminina, por causarem alterações na mudança dos níveis hormonais e diminuirem a libido.

Com relação à hipertensão arterial sistêmica, 91,7% dos casos estudados referiram não possuir essa doença. Apesar de a obesidade estar relacionada com diversas comorbidades, como a própria hipertensão arterial, o *diabetes mellitus* tipo 2 e alguns tipos de câncer e dislipidemias,^( 26 )^há pacientes obesas que apresentam apenas uma ou nenhuma destas comorbidades. Isto não significa que existam casos de obesidade saudável, pois o processo inflamatório é inerente a esta doença, causando diversos danos à saúde dos pacientes.

O resultado de atividade física, com poucas mulheres sedentárias (nenhuma no Grupo Caso e quatro – 14% − no Controle), pode ter relação com a forma como o IPAQ classifica as atividades moderadas, incluindo atividades domésticas que aumentam moderadamente a respiração ou os batimentos do coração e foram bastante incluídas pelas participantes desta pesquisa como a única atividade física relatada. O potencial do presente estudo foi demonstrar que a população estudada segue o mesmo padrão encontrado em pesquisas de outros países,^( 7 , 11 )^reforçando a hipótese de que a obesidade interfere na infertilidade.

## CONCLUSÃO

A obesidade influencia na infertilidade, ou seja, há correlação positiva entre a obesidade e a infertilidade. Os conhecimentos sobre tal relação são importantes para aumentar as chances das mulheres subférteis de reverterem a situação de infertilidade e terem uma gravidez saudável. Melhorias no hábito alimentar e prática de exercício físico são importantes para promover mudanças na composição corporal e no estado nutricional destas mulheres.

O melhor manejo de mulheres com anovulação crônica deve ser o desenvolvimento de estratégias que estimulem o controle do peso, antes de se começar um ciclo de tratamento de reprodução assistida.

Sugere-se que os hospitais que fornecem tratamento para infertilidade ofereçam atendimento nutricional e incentivem a prática de exercício físico à população assistida.
